# Measurement of temperature and density using non-collective X-ray Thomson scattering in pulsed power produced warm dense plasmas

**DOI:** 10.1038/s41598-018-26608-w

**Published:** 2018-05-30

**Authors:** J. C. Valenzuela, C. Krauland, D. Mariscal, I. Krasheninnikov, C. Niemann, T. Ma, P. Mabey, G. Gregori, P. Wiewior, A. M. Covington, F. N. Beg

**Affiliations:** 1 Center for Energy Research, University of California, San Diego, California, 92093-0417 USA; 2 Department of Physics and Astronomy, University of California, Los Angeles, California, 90095 USA; 3 Lawrence Livermore National Laboratory, Livermore, California, 94550 USA; 4 Department of Physics, University of Oxford, Parks Road, OX1 3PU Oxford, United Kingdom; 5 Nevada Terawatt Facility, Department of Physics, University of Nevada Reno, Reno, Nevada 89557 USA

## Abstract

We present the first experimental measurement of temperature and density of a warm dense plasma produced by a pulsed power driver at the Nevada Terawatt Facility (NTF). In the early phases of discharge, most of the mass remains in the core, and it has been challenging to diagnose with traditional methods, e.g. optical probing, because of the high density and low temperature. Accurate knowledge of the transport coefficients as well as the thermodynamic state of the plasma is important to precisely test or develop theoretical models. Here, we have used spectrally resolved non-collective X-ray Thomson scattering to characterize the dense core region. We used a graphite load driven by the Zebra current generator (0.6 MA in 200 ns rise time) and the Ti He-α line produced by irradiating a Ti target with the Leopard laser (30 J, 0.8 ns) as an X-ray probing source. Using this configuration, we obtained a signal-to-noise ratio ~2.5 for the scattered signal. By fitting the experimental data with predicted spectra, we measured T = 2±1.9 eV, ρ = 0.6±0.5 gr/cc, 70 ns into the current pulse. The complexity of the dense core is revealed by the electrons in the dense core that are found to be degenerate and weakly coupled, while the ions remain highly coupled.

## Introduction

High Energy Density (HED) plasmas are frequently produced in pulsed power systems either by plasma compression using the intense Lorentz force (***J***X***B***) or when the plasma starts from solid, metallic materials (such as wire arrays or liners) where the phase state of the material changes from Warm Dense Matter (WDM) up to a plasma state as the current Ohmically heats the material^1^. Although dense matter is usually difficult to diagnose, the high temperatures achieved in the former case help to produce copious amounts of radiation, shedding light on the plasma properties. On the other hand, the latter case remains almost undiagnosed due to its low temperatures (<10 eV), and because the wire material moves in the phase space in a complex way^1,2^ as the magnetic pressure and plasma pressure change in time. In general, accurate knowledge of the transport coefficients as well as the thermodynamic state of the plasma is required to precisely test or develop theoretical models (e.g. electrothermal instability^3^) which have been difficult to achieve in the case of the current heated wire for the reasons aforementioned. Furthermore, the exact plasma formation time (which is very important in pulsed power systems) depends on the total energy deposition in the material; which is a function of the trajectory of the electrical conductivity in time. Therefore, it is important to have an experimental technique that allows us to look at the very beginning of the current-heated metallic loads and measure temperature (T), density (ρ), and ionization state (Z). One possibility is X-ray Thomson scattering^4^.

X-ray Thomson scattering (XRTS) is a powerful experimental technique for diagnosing WDM. This technique has become common in kilojoule-class laser facilities where multiple beams can readily create plasmas as well as X-ray sources^5–10^. For example, XRTS was used in ref.^8^ to observe plasmon scattering spectra in near solid-density matter and infer the electron density, and in ref.^11^ strong ion-ion correlation was measured in shock-compressed aluminium. Also, with recent advances in free electron laser technology, X-ray sources with small enough bandwidth have become available, allowing the investigation of the low-frequency ion modes in dense matter. For example, the Linac Coherent Light Source has been utilized to resolved ionic interactions at atomic scale lengths and determine their physical properties. Measurements have resolved the transition to WDM and demonstrated that short-range repulsion between ions must be accounted for to obtain accurate structure factors and equation of state data^12^. In another work, highly resolved measurements of the plasmon spectrum in isochorically heated aluminium targets was obtained^13^; detailed balance was used to obtain temperature from the plasmon features and also the electrical conductivity was measured from the plasmon damping.

Pulsed power facilities on the other hand, require an additional capability to create a separate X-ray source while driving the HED system. At present, only a few facilities in the world exist that couple a high-intensity laser to a pulsed power driver, thus limiting the implementation of this technique on pulsed power facilities. One such facility is Sandia National Laboratories, where in a recent work on the Z pulsed power generator, space-resolved X-ray Thomson scattering from shocked carbon foams was accomplished^14^. Carbon foams were shocked by using the flyer plate technique and the Z-beamlet laser was used to produce 6.2 keV X-ray from a Mn foil. The goal of the work was to enable the advances of WDM physics by combining the powerful XRTS diagnostic with the extreme environment created at the Sandia Z pulsed power accelerator. Another Facility capable of coupling a high intensity laser to a pulsed power generator is the Nevada Terawatt Facility (NTF), where the work presented here was carried out.

**Figure 1:**
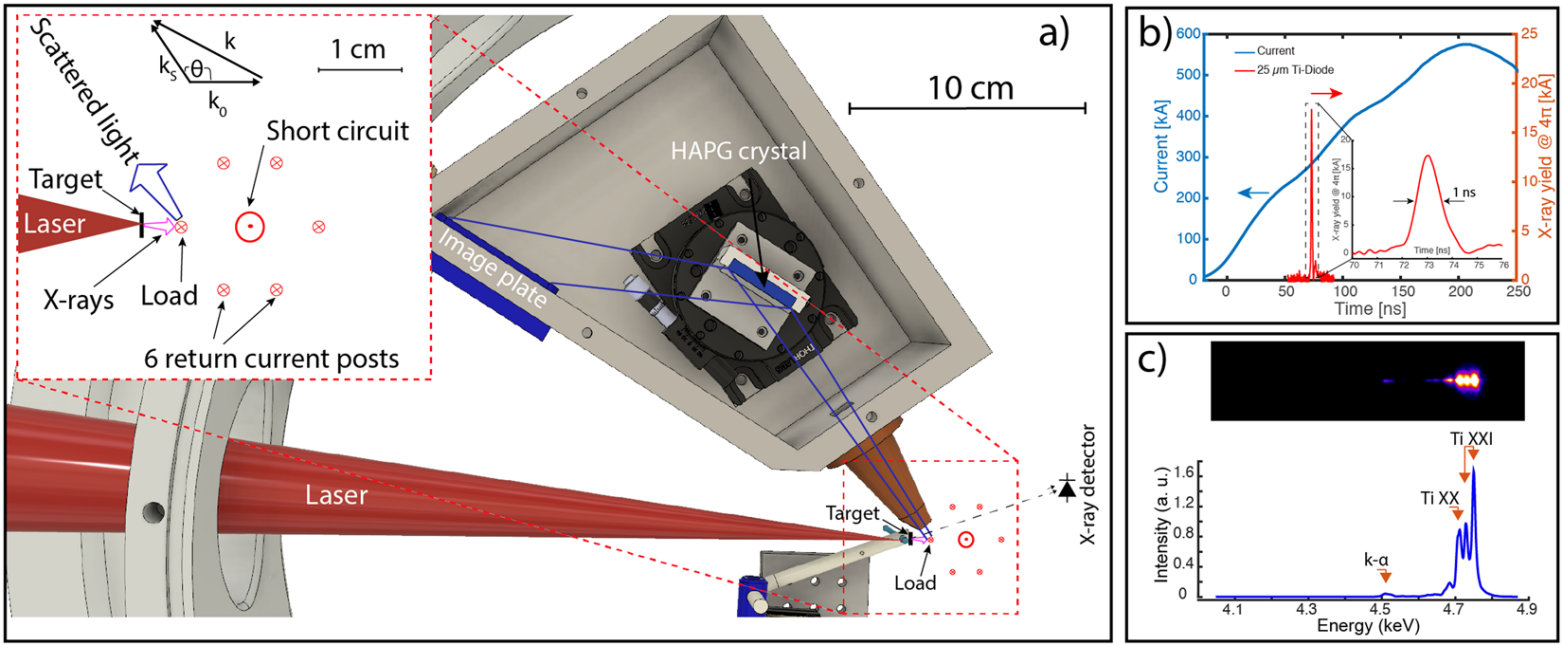
(**a**) X-ray Thomson scattering experimental arrangement at the NTF. The Leopard laser (30 J, 0.8 ns) produces Ti He-α emission from a titanium target and interacts with the graphite load heated by the Zebra current driver. The insert shows a close-up view of the target load region: the total current is carried by the central rod (short circuit) and then it splits in six return current post, the graphite load is located in one of them. (**b**) characteristic Ti-filtered diode signal obtained in the experiment, 1 ns X-ray pulse width was measured. (**c**) Raw spectrometer data from the Ti X-ray source recorded on the IP (top) and horizontal lineout of the data (bottom).

In XRTS, incident radiation with wave vector **k**_**0**_ interacts with the plasma and the detector viewing from an angle θ observes the scattered light with wave vector **k**_**S**_ (see insert in Fig. 1a)). The scattering wave vector **k** in the non-relativistic limit (or small momentum transfer) is defined as:

1 $${\boldsymbol{k}}={{\boldsymbol{k}}}_{s}-{{\boldsymbol{k}}}_{0},\,k=|{\boldsymbol{k}}|=4\pi \frac{{E}_{0}}{hc}\,\sin (\frac{\theta }{2})$$

and determines the amount of momentum transfer in the scattering process ($\hslash {\boldsymbol{k}}$). Here E_0_ is the probing photon energy, *h* ($\hslash =h/2\pi$) is the Plank’s constant and *c* is the speed of light. By comparing the scale length of the electron density fluctuation accessed by Thomson scattering (~1/k) with the electron screening length ${\lambda }_{s}$, the scattering parameter alpha is defined : $\alpha =1/k{\lambda }_{s}$. Where the screening length is given by λ_s_ = (n_e_e^2^/ε_0_k_B_T_cf_)^1/2^, and T_cf_ = (T_e_^2^ + T_q_^2^)^1/2^ is the effective temperature, T_q_ = ε_F_/(1.3251–0.1779√r_s_), r_s_ = d/a_B_, and a_B_ is the Bohr radius. This approach was shown to reproduce the finite-temperature static response of an electron fluid, valid for any degeneracy^15,16^. For the nondegenerate case, it gives the classical Debye length and for the degenerate system it gives the Thomas-Fermi screening length. Depending on the relation of the alpha parameter to unity, two scattering regimes are attainable. When α < 1, called non-collective regime, the probing scale-length is smaller than the screening length, and individual electrons are resolved. Two peaks are observed in this regime, the Rayleigh peak due to bound electrons (at the original photon energy) plus the red-shifted Compton peak due to kinematically free electrons (truly free and weakly bound). The Compton term is broadened by the Doppler effect due to the velocity distribution of the particles. Therefore, the electron distribution function (Boltzmann or Fermi-Dirac) could be measured, and density and temperature can be acquired. When α > 1, it defines the collective scattering regime and fluctuation at a scale length larger than the screening length are observed. Here, the scattering signal is composed of 5 peaks^17,18^, two are related to photons scattering off electron-plasma waves, other two are associated to ion-acoustic waves and there is also a central Rayleigh peak. The collective regime carries information about collisional processes and transport coefficients in the plasma, thus electrical and thermal conductivity can be obtained in this regime^19^.

In this letter, we present the first measurement of the plasma density and temperature of the initial phase of a current-heated wire using non-collective XRTS at the Nevada Terawatt Facility.

## Experimental description and Results

One of the stringent requirements for XRTS is a bright X-ray source, which in a pulsed power environment could be obtained by placing a second Z-pinch load in the current diode, e.g. an X-pinch^20^, or an independent source such as a laser-produced X-ray source. The latter case is clearly a more attractive solution as the timing and location of the source can be controlled independently of the current driver. The experiment presented here utilized the NTF’s mega-Ampere current driver Zebra and the high intensity Leopard laser. Zebra was operated in its long pulse mode, delivering 0.6 MA in 200 ns to the load. The Leopard laser provided 30 J in 0.8 ns at 1057 nm to generate the X-ray source.

Figure 1a) shows the setup used for XRTS experiments. The laser interacts with the Ti foil and produces Ti He-α X-rays that propagate towards the plasma load driven by the Zebra driver. A Highly Annealed Pyrolytic Graphite crystal (HAPG) collects backscattered photons at an angle of θ = 100° (see insert in Fig. 1a). We placed the load in one of the six return current posts (closer to the X-ray source) to increase the number of photons on the plasma load as well as to optimize the scattered signal. Thus, the load sees a fraction of the total current (see Fig. 1b)). We did not measure the current directly in the graphite load. Assuming the current distribution is symmetric, the load current should be 1/6th of the total current. In the main load region, we used a short circuit that does not produce plasma or X-rays (to reduce the background noise). With this configuration, the Ti foil was placed 5 mm apart from the plasma load, which increased the X-ray intensity by almost 100 times compared to the intensity if we had placed the load at the center. The insert shown in Fig. 1a) represents the load arrangement. The short circuit is represented by the arrow tip in the concentric geometry while the return current posts are represented by crosses equally spaced around the center. The load investigated was a graphite foil, 3 mm wide by 5 mm long and 400 μm thick. Graphite was chosen because the scattering volume is larger at 4.75 keV photons compared to higher atomic number materials—such as Al—normally used in these kinds of experiments. It also helped to decrease the background signal on the image plate (IP) produced by the interaction of fast electrons (generated by the laser-target interaction) with the plasma load, which increases with atomic number. Scattered X-rays with wave vector **k**_**S**_ are observed at an angle θ = 100° ± 17° with respect to the incident X-rays (with wave vector **k**_**0**_) and the scattering wave vector **k** is given by Eq. 1.

We characterized the Ti He-α emission at 4.75 keV as an X-ray probing source using the Leopard laser by using a setup similar to that shown in Fig. 1a) for XRTS experiments, but with the plasma load removed and the spectrometer pointing directly towards the X-ray source (keeping the Bragg angle the same). This provided both a measurement of the He-α line width and a calibration point for comparison of brightnesses on data shots. The absolute Ti He-α yield was measured using absolutely calibrated filtered diodes^21^. The diodes looked for forward emission on data shots, and their location were consistent between characterization experiments and XRTS experiments (See Fig. 1a)). We used 18 μm scandium, 25 μm titanium and 14 μm vanadium filters to obtain a good estimate of the laser to He-α conversion efficiency.

With this filter configuration, the contributions from K-α, He-α and H-α emission could be measured. Figure 1b) shows the characteristic diode signal obtained during the experiments using the Ti-filtered diode. Comparing the 3-filter diode signals (not shown here), we conclude that the dominant contribution is from He-α and that the temporal emission width is ~1 ns, which is much shorter than the time scale of the plasma load evolution (of the order of the driver rise time ~200 ns, see current trace in Fig. 1b). We varied the Ti target location from best laser focus to find the optimal laser intensity for conversion of laser energy into He-α emission, and determined that ~10^15^ W/cm^2^ is the optimum value. We also performed a foil thickness scan and found an optimum value of 2 μm. From the diodes’ signals, we measured a total He-α energy of 15.4±0.8 mJ, equivalent to (2.0 ± 0.1)×10^13^ photons over 4π steradians (considering homogeneous emission), corresponding to 0.043 ± 0.002% laser conversion efficiency into He-α photons. Calculations showed that this number was barely enough for XRTS experiments when optimum collection efficiency and extremely good background X-ray shielding is considered. The raw spectrometer data in Fig. 1c) shows He-α is the dominant line, but it also shows the intercombination and satellite lines, limiting the bandwidth. Including these lines, we obtain ~1% bandwidth, which is the required value for the non-collective Thomson scattering regime. We also confirm that K-α emission is not significant at this laser intensity.

Given this configuration –with scattering angle of 100° and central scattering X-ray energy of 4.75 keV— we obtained a scattering parameter of α = 0.4, and therefore conclude that the scattering process is in the non-collective regime. The Compton shift is 52 eV and it should be resolved given the probing X-ray bandwidth.

The plasma collection solid angle is ~0.6 sr, thus the collection fraction is 0.6/4π ~0.05 and the number of photons arriving on plasma is then N_p_~1 × 10^12^. The fraction of scattered photons is given by ${F}_{S}={\sigma }_{Th}{N}_{e}l$, where σ _Th_ = 6.65 × 10^−25^ cm^2^ is the Thomson cross section, *N*_*e*_ is the electron density and *l* is the path length (*l* ~100 μm). Therefore, *F*_S_ = 0.002 and the number of scattered photons is *N*_*S*_ = *F*_*S*_*N*_*p*_~ 2 × 10^9^. From these, the number of counts produced in the IP detector will be:

2 $${N}_{c}={N}_{S}\frac{{{\rm{\Omega }}}_{cryst}}{4\pi }{\eta }_{det}{R}_{crys}$$

where Ω_cryst_ is the crystal collection solid angle, R_crys_ is the crystal reflectivity and η_det_ is the detector efficiency. In our case Ω_cryst_/4π~4.8 × 10^−5^, R_crys_~0.5 and η_det_~0.17. So, N_c_~1 × 10^4^ counts, which is ~3 times larger than the lowest background signal achieved during the experiments.

**Figure 2:**
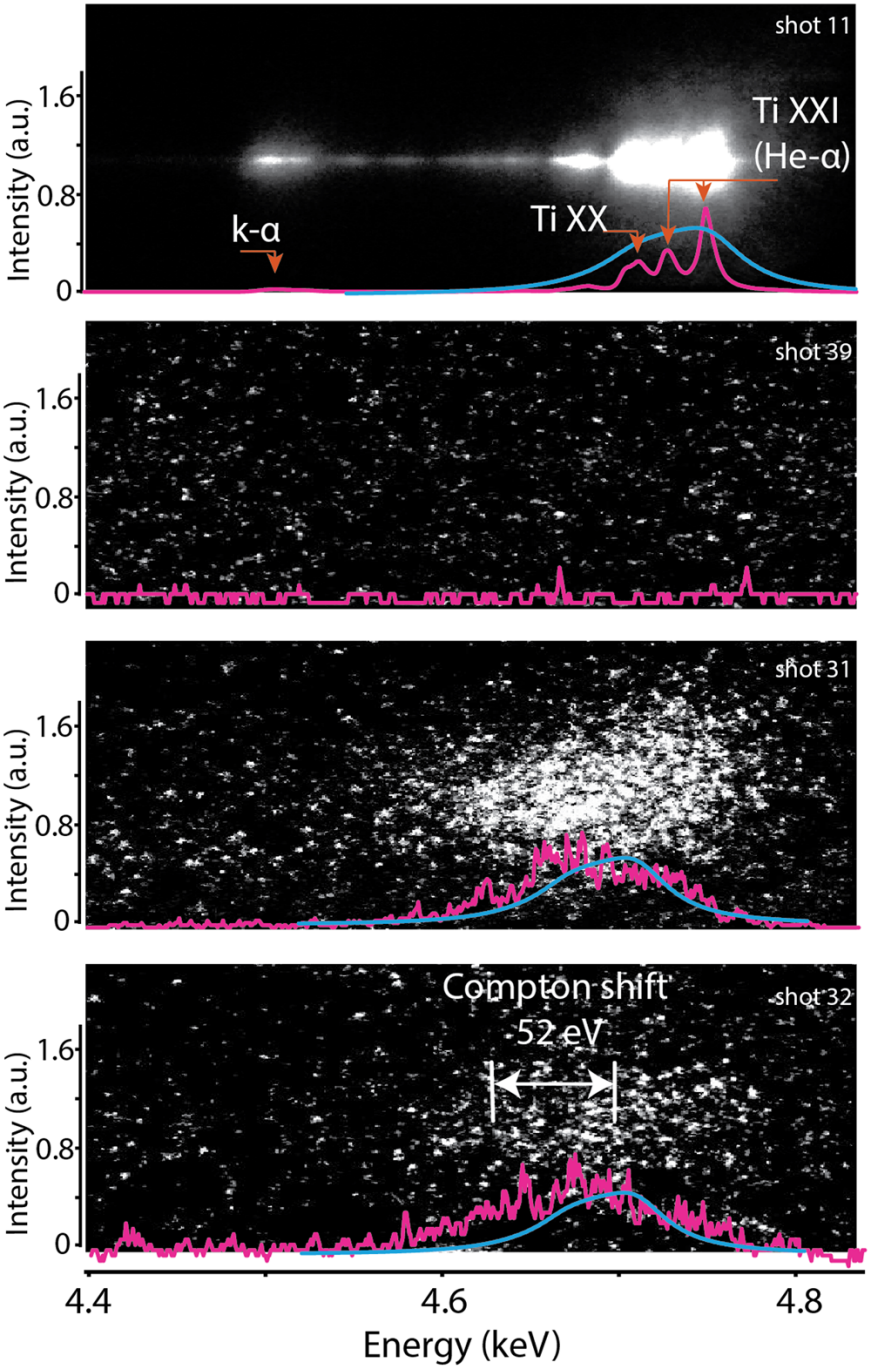
Raw spectrometer data showing (**a**) source He-α line emission (**b**) background shot from Zebra only (Leopard was not fired) (**c**) scattering from a cold load (Zebra was not fired) and (**d**) scattering from the current-heated load (both Zebra and Leopard were fired). The pink line is a lineout of the data and the blue line is the effective source profile.

Figure 2 displays a comparison of raw spectrometer data from several run configurations: (a) He-α emission with the spectrometer pointed towards the Ti target, (b) Zebra driver only, confirming no signal at the He-α location coming from the graphite load, (c) the graphite load in place but the laser only was fired, showing a feature at the He-α above the background level and indicating scattering off of the cold graphite load and (d) Leopard laser was fired 70 ns into the Zebra current pulse (see Fig. 1b)). As in the cold target case, we also see signal at the He-α position, above the background noise, suggesting it is scattered light. Notice that we do not observe the emission lines because the source is convolved with the plasma size, which is shown by the light blue line in Fig. 2a). If we compare the scattered signal (for both cold and heated load) with the effective source profile (calculated given the plasma source size and spectrometer resolution), we observe that the cold target is almost fitted by the effective source profile, however, the scattered signal for the current heated load is broadened, indicating plasma heating and also that the shift is consistent with the Compton energy shift ${\hslash }^{2}{k}^{2}/2{m}_{e}$~52 eV. The effective source profile shown in light blue color in Fig. 2 was obtained by assuming every point in the load radiates as the original X-ray source (pink trace in Fig. 2a). The final profile is then found by adding them up numerically and considering the relative energy shift on the detector as the emitters change position in the load.

### Analysis and Discussion

The analysis of scattering data is complex and models are usually developed to compute the total cross-section to make a comparison of synthetic spectra with the experimental data. The total scattering cross section is given by the following expression^16^:

3 $$\frac{{\partial }^{2}\sigma }{\partial {\rm{\Omega }}\partial \omega }={\sigma }_{T}\frac{{k}_{S}}{{k}_{0}}S(k,\omega )$$

where, σ_T_ = 6.65 × 10^−25^ cm^2^ is the Thomson cross section and *S*(*k*,*ω*) is the dynamic structure factor, which is a function of the scattering wave-vector **k** and frequency ω of scattered light. *S* carries information about the plasma parameters. It can be written as^22^:

4 $$S(k,\omega )={|{f}_{I}(k)+q(k)|}^{2}{S}_{ii}(k,\omega )+{Z}_{f}{S}_{ee}^{0}(k,\omega )+{Z}_{c}{\int }^{}{S}_{ce}(k,\omega -\omega \text{'}){S}_{s}(k,\omega \text{'})d\omega \text{'}$$

where the first term reflects the electrons that follow the ion motion, including both bound electrons (represented by *f*_*I*_*(k)*), and the screening electron cloud (represented by *q(k)*); it gives rise to the ion feature or elastic term in the scattering signal. The second term is the contribution from kinematically free electrons that do not follow the ion motion (represented by ${Z}_{f}$) and gives the thermal broadened Compton signal. It is important to distinguish between electrons that are kinematically free with respect to the scattering process and core electrons that are tightly bound to the atom. The kinematically free electrons includes both free electrons (removed from the atom by ionization) and the valence (weakly bound) electrons, thus *Z*_*f*_ = *Z* + *Z*_*v*_, where *Z* is the number of electrons removed from the atom and *Z*_*v*_ is the number of valence electrons. This condition is equivalent to assuming that the energy transfer to the electrons by Compton scattering is larger than the binding energy. The last term in Eq. 4) is the inelastic scattering by bound electrons, such as Raman scattering, and it is represented by ${Z}_{c}$.

In order to calculate the total scattering cross-section, we assume that the electron and ion temperatures are equal in these strongly collisional states. To calculate the ion-ion density correlation function (*S*_*ii*_(k,ω)) we make the approximation *S*_*ii*_(k,ω) = *S*_*ii*_(k)δ(ω) as we cannot currently access this low frequency part of the spectrum. Thus, we only calculate the static structure factor for ion-ion correlations *S*_*ii*_(k). We also assume that the ions are nondegenerate (ratio of thermal energy over Fermi energy) and the electrons obey the Fermi-Dirac distribution function. Due to the scaling of the Coulomb interactions with the ionic charge, it is possible to create strongly coupled (Γ_ii_ ≫ 1) WDM states but with Γ_ee_ ~1, where the coupling parameter Γ is defined as the ratio between intra-particle potential energy over particle’s kinetic energy. This means a highly coupled theory is necessary to describe the microscopic response of the ions, the electrons on the other hand can be modeled using intermediate techniques. Under these conditions and within the framework of the screened one-component plasma approximation (SOCP) we can calculate *S*_*ii*_(k) using a semiclassical approach that takes quantum effects into account^23^. The free electron density-density correlation function (${S}_{ee}^{0}$(k,ω)) can be calculated through the fluctuation-dissipation theorem under the assumption that interparticle interactions are weak so that nonlinear interactions between different fluctuations are negligible. The dielectric function is then derived in the random phase approximation (RPA). More details of the model and calculation can be found in the detail in refs.^16,23^. The model has been successfully implemented to analyze XRTS experiments and it correctly reproduces the Doppler-broadened and Compton-shifted scattered spectra (see for example ref.^5^).

**Figure 3:**
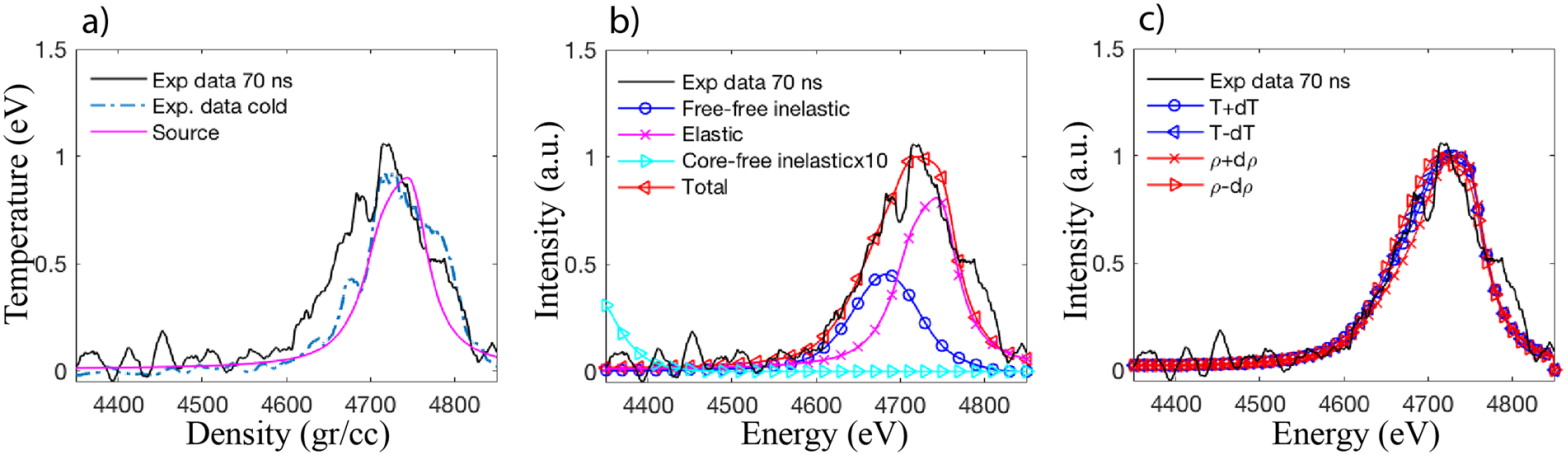
(**a**) Comparison of the cold and driven data together with the source profile, showing the signal broadening of the driven case due to Compton scattering and plasma heating. (**b**) the best match: ρ = 0.6 gr/cc, T = 2 eV, Z_f_ = 4 is plotted against the experimental data. The different scattering contributions are also shown: elastic, free-free inelastic and bound-free inelastic. (**c**) Estimate of the error bars is found by varying the theoretical spectra within the fluctuation of the data, we find that ρ = 0.6 ± 0.5gr/cc and T = 2 ± 1.9 eV, the large error is mainly given by the small signal-to-noise ratio.

Figure 3 shows the result of the data analysis. First, in Fig. 3a) we compare the cold and driven load case together with the source profile, where we clearly see the broadening in the driven case due to the increase in plasma temperature. The driven case was fitted by adjusting three input parameters: temperature (T), mass density (ρ), and the number of free electrons (Z_f_). In our case, we have chosen Z_f_ = 4 as the Compton energy shift (52 eV) is larger than the L-shell binding energy (~10 eV), in this case both free and valence electrons behave as single particles and differences in their momentum distribution provide negligible differences in the scattering spectra^24,25^. We generated synthetic spectra varying T, ρ and tried to find a set of parameters that best matches the data. We performed a χ^2^ (chi-squared) analysis where we try to minimize the error between the experimental data and synthetic spectra produced by the model (not shown here). We found that the error is minimum for T = 2 eV, ρ = 0.6 gr/cc. In Fig. 3b) we compare the best fit with the scattering data; the different scattering contributions are also shown: elastic, free-free inelastic and bound-free inelastic. The free-free inelastic term includes both the contribution from truly free electrons and valence electrons, while the bound-free term is the contribution from core electrons that are tightly bound to the atom. We observe that the bound-free contribution is not significant and the scattered spectrum is explained by the free-free term only. Notice the scattering signal presented in Fig. 3b) is an average of two shots at the same time (~70 ns) to increase the signal to noise. We can determine the accuracy of these values by varying the theoretical spectra within the fluctuation of the data. We find the error for density is ±0.5 g/cc and temperature is ±1.9 eV, both shown in Fig. 3c). The accuracy is mainly determined by the signal-to-noise ratio of the scattered signal (~2.5), which was limited by the low X-ray yield obtained with Leopard laser (~30 J). Notice that ~10% accuracy is standard nowadays for XRTS measurements with higher X-ray yield sources^5,10^.

From these values, we observe that the electrons are degenerate Θ_e_~0.2 and weakly coupled Γ_ee_~1, which is within the range of validity of the model utilized to calculate the non-elastic scattering spectra^16^; while the ions are highly coupled Γ_ii_~5 and non-degenerate. The SOCP model used to calculate the elastic term is valid as long as the system is in a liquid or plasma state and the ion coupling is not too high. If we use the Lindeman’s law^26^ to estimate the melting temperature for carbon at ~0.6 gr/cc we get a melting temperature of at least an order of magnitude lower than the plasma temperature; as Γ_ii_~5, this assures the model is valid. Nevertheless, the applicability of the SOCP model may be borderline at the low temperature end of the error estimation (0.1 eV, Γ_ii_~36).

As it was mentioned above Z_f_ is not the true ionization state but the number of electrons that participate in the Thomson scattering process. It includes weakly bound electrons (binding energy <energy transfer by Compton scattering) and truly free electrons. This value should be considered as an upper limit of the true ionization state. As an example, if we consider the Thomas-Fermi model^27^ to estimate the ionization state given the plasma temperature and density found above, we obtain Z_TF_~1.2, which is smaller than the value used here. Note that according to the Thomas-Fermi model, plasma ionization is mainly due to pressure ionization at the measured plasma temperature and density.

### Concluding Remarks

We have presented the first experimental results on XRTS at the Nevada Terawatt Facility demonstrating its viability as a diagnostic for use on mega-Ampere scale Z-pinch loads. Experiments were challenging, mainly because of the low laser energy (~30 J) used to produce the X-ray probing source. Furthermore, the Zebra current generator produces significant background radiation perturbing the measurement. However, we managed to observe the spectral shift to lower energies in the driven case, see for example Fig. 3a), compared to the cold case.

Broadening on the scattered signal in the driven case indicates plasma heating. Fitting the experimental data with predicted spectra, we were able to extract the plasma temperature (T = 2 ± 1.9 eV) and density (ρ = 0.6 ± 0.5 gr/cc)). Where the error bars are determined by the signal-to-noise ratio of the scattered signal (~2.5), which was limited by the low X-ray yield obtained with Leopard laser.

Even though the results presented here are for a single point in time during the evolution of the plasma, it can be easily extended to different times into the current pulse, allowing us to explore the full transition from solid matter into plasma.

The study of WDM in pulsed power has been hindered by the lack of available diagnostics. For example, transport coefficients in the region of metal-insulator transition and in the vicinity of the critical point are not well understood and the thermodynamic parameters are not usually measured directly, but calculated based on equation of state (EOS) databases. So, as it is demonstrated here, XRTS is able to access the dense core of a current heated plasma and gives the thermodynamic parameters as a function of time. We believe this work should be seen as baseline for future work to properly interpret laboratory measurement and model validation of plasma microscopic properties such as electrical conductivity, thermal conductivity, EOS, and opacity.

## Methods

### Laser and target

The Leopard laser was used in its long pulse mode (~30 J, 0.8 ns) to produce titanium He-α X-rays (4.75 keV). The laser beam was focused to a ~60 µm diameter spot by a f/# ~ 8 lens placed outside the vacuum chamber, giving an on-target intensity greater than 10^15^ Wcm^−2^ at best focus. The Ti target was glued onto a glass stalk (3 mm diameter) and mount into a 3-axis linear stage. The intensity scanned on the titanium target was done by moving the linear stage towards the laser and looking at the filtered diodes signal for best He-α emission. We used 2, 6 and 20 μm Ti foil targets to study the effect foil thickness on X-ray yield.

### X-ray shielding and detection

Both the Zebra current driver and the laser-target interaction produce significant amount of background noise, and additional shielding to the 0.5″ thick aluminium walls (shown in Fig. 1a)) of the crystal housing had to be used. A combination of 10 mm lead outside the box and 3 mm of copper plus 20 mm of C3H6 plastic inside the box gave us two orders of magnitude lower background. The image plate was also embedded inside the Al wall to increase the areal mass density between the target-load and the image plate (see Fig. 1a)). Ti He-α yield was measured using absolute calibrated filtered diode from optodiode, part number AXUVHS5. The diodes looked for forward emission and they remained at that location for the XRTS experiments. We used 18 μm Sc, 25 μm Ti and 14 μm V filters to obtain a good estimate of the laser to He-α conversion efficiency – V filter gives the contribution from k-α emission, Ti from k-α + He-α and SC from k-α + He-α + H-α, so from the three-filter set the individual contribution can be estimated. The spectrometer used a Highly Annealed Pyrolytic Graphite crystal (HAPG) from Optigraph^28^. The crystal was bent in the sagittal direction in order to be used in the Von Hamos configuration. The Bragg angle for 4.75 keV is 23° and unit magnification was used to satisfy mosaic focusing. Fujifilm MS image plate and FLA-5000 scanner were used to read and record the data. Appropriate filters were added in the spectrometer: 5 μm Ti + 13 μm kapton at the spectrometer tip and 13 μm kapton in front of the image plate. The Ti filter gave us a transmission window ~2.3keV-5keV to see the scattered light and the kapton filter helps to protect the spectrometer and image plate of debris coming from the plasma. The 13 μm kapton filter in front of the image plate also blocked the low energy fluorescence light produce inside the spectrometer.

### Plasma load

The load consisted of a graphite strip, machined out of a 1.8 gr/cc graphite block. Graphite was chosen because the scattering volume is larger at 4.75 keV photons compared to higher atomic number materials—such as Al—normally used in these kinds of experiments. It also helped to decrease the background signal on the IP produced by the interaction of fast electrons (generated by the laser-target interaction) with the plasma load, which increases with atomic number.
